# The Genetic Mechanisms Driving Diversification of the *KIR* Gene Cluster in Primates

**DOI:** 10.3389/fimmu.2020.582804

**Published:** 2020-09-11

**Authors:** Jesse Bruijnesteijn, Natasja G. de Groot, Ronald E. Bontrop

**Affiliations:** ^1^Comparative Genetics and Refinement, Biomedical Primate Research Centre, Rijswijk, Netherlands; ^2^Theoretical Biology and Bioinformatics, Utrecht University, Utrecht, Netherlands

**Keywords:** Killer cell immunoglobin-like receptor, KIR, NK cell, NK cell education, human, macaque, non-human primates

## Abstract

The activity and function of natural killer (NK) cells are modulated through the interactions of multiple receptor families, of which some recognize MHC class I molecules. The high level of *MHC class I* polymorphism requires their ligands either to interact with conserved epitopes, as is utilized by the NKG2A receptor family, or to co-evolve with the MHC class I allelic variation, which task is taken up by the killer cell immunoglobulin-like receptor (KIR) family. Multiple molecular mechanisms are responsible for the diversification of the *KIR* gene system, and include abundant chromosomal recombination, high mutation rates, alternative splicing, and variegated expression. The combination of these genetic mechanisms generates a compound array of diversity as is reflected by the contraction and expansion of *KIR* haplotypes, frequent birth of fusion genes, allelic polymorphism, structurally distinct isoforms, and variegated expression, which is in contrast to the mainly allelic nature of MHC class I polymorphism in humans. A comparison of the thoroughly studied human and macaque *KIR* gene repertoires demonstrates a similar evolutionarily conserved toolbox, through which selective forces drove and maintained the diversified nature of the *KIR* gene cluster. This hypothesis is further supported by the comparative genetics of *KIR* haplotypes and genes in other primate species. The complex nature of the *KIR* gene system has an impact upon the education, activity, and function of NK cells in coherence with an individual’s MHC class I repertoire and pathogenic encounters. Although selection operates on an individual, the continuous diversification of the *KIR* gene system in primates might protect populations against evolving pathogens.

## Introduction

**FIGURE 1:**
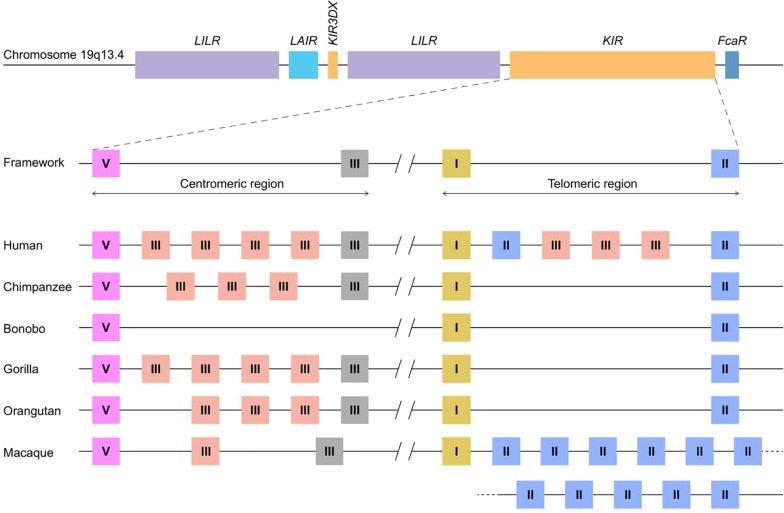
KIR haplotype organizations in different primate species. A schematic overview of the Leukocyte Receptor Complex (LRC) on chromosome 19q13.4 and KIR haplotype organizations in different primate species. A fixed copy of the *KIR3DX* gene is located within the primate *LILR* gene cluster, whereas the expanded *KIR* gene cluster is flanked by the *LILR* and *FcaR* genes. The expansion involved four different *KIR* gene lineages – I, II, III, and V – the members of which are indicated as yellow, blue, red/gray, and pink boxes, respectively. The gray lineage III boxes represent pseudogenes. In most hominoids, KIR haplotype organizations follow a standard framework, in which the centromeric and telomeric regions are bordered by genes from lineages V and III, and lineages I and II, respectively. A relatively large non-coding segment separates the centromeric and telomeric haplotype sections.

The innate and adaptive arms of the immune system are interconnected, and feature several effector functions that provide efficient and specific protection against infection and tumor formation. Major components of the adaptive arm comprise T and B lymphocytes characterized by rearranging antigen receptors, which exert cytotoxic and humoral immunity, respectively. The cytotoxicity mediated by T lymphocytes highly depends on the presentation of intracellular antigen segments derived from pathogens by MHC class I molecules and subsequent clonal expansion of cells with specific receptors. A third type of lymphocytes bridge the innate and adaptive immune response, and comprises natural killer (NK) cells, which participate, for instance, in the recognition and elimination of aberrant cells that down-regulate their MHC class I expression to evade detection by T lymphocytes (1). Without prior priming or clonal expansion, inhibitory and activating receptors on the NK cell surface interact with MHC class I molecules on nucleated cells to modulate NK cell effector functions, which include the killing of target cells by the release of cytolytic proteins and the regulation of other immune cells by the secretion of cytokines (2). The genes encoding the MHC class I molecules are considered the most polymorphic genes known in vertebrates, a phenomenon that resulted from selective pressure to adapt to the rapid diversification of pathogens. This extended repertoire of *MHC class I* genes and alleles requires the NK cell receptors to co-evolve to maintain a functional relation with their ligands. The recognition of MHC class I molecules by NK cells involves two receptor families: the conserved CD94:NKG2A receptors and the highly polymorphic and diverse killer cell immunoglobulin-like receptors (KIR). Both receptor families consist of inhibitory and activating members. Their engagement with MHC class I molecules calibrates the responsiveness of NK cells through a continuous educational process, which largely controls subsequent NK cell activity (3, 4). The KIR receptors are encoded within the Leukocyte Receptor Complex (LRC) on chromosome 19q13.4, and share this genomic region with other structurally similar immune-regulators, such as the leukocyte Ig-like receptors (LILRs) and the leukocyte-associated Ig-like receptors (LAIRs; Figure 1) (5). Based on different *Alu* elements that can be regarded as a molecular clock, the initial expansion of the primate *KIR* gene cluster is estimated to date back to approximately 31 to 44 million years ago. This process continued, and is currently reflected by extensive gene duplications and point mutations (6). Different diversifying mechanisms in combination with evolutionary selective factors propel the complex *KIR* gene content at the individual level but also at the population and species-specific level, which all together contribute to the heterogeneity of NK cell subsets and their activity. The *KIR* gene diversification is not limited to humans. Comparative analyses that include other primate species might help in gaining a thorough understanding of the evolutionary processes that resulted in the diversification of this gene system. In the following sections, we will discuss the different genetic mechanisms that drove the evolution of the highly plastic *KIR* gene system in hominoids (humans and great apes) and Old World monkeys, and how this might influence their NK cell response.

## Co-Evolution of *MHC* and *KIR* Genes

The complex *KIR* gene system requires a comprehensive nomenclature guideline for the different genes and allotypes in order to distinguish the corresponding receptors by their structure and signaling potential (7–9). Receptors may contain one to three Ig-like domains, which are encoded by exon 3 (D0 domain), exon 4 (D1 domain), and exon 5 (D2 domain), and are referred to as KIR1D, KIR2D, and KIR3D in the official nomenclature. Further classification defines the inhibitory or activating signaling function of the KIR receptors, which is characterized by either a long or short cytoplasmic tail, respectively, and specified with an “L” or an “S” following the domain number denotation. The long cytoplasmic tail contains one or two immune tyrosine-based inhibitory motifs (ITIMs), whereas the signal transduction of activating KIR depends on the interaction with an adaptor molecule that includes an immune tyrosine-based activating motif (ITAM) such as DAP12. Pseudogenes are indicated with a “P” (e.g., *KIR3DP*). In addition, a four-character species designation is included in front of the KIR acronym (e.g., *Mamu-KIR3DL20* in rhesus macaques; *Macaca mulatta*).

The mammalian *KIR* genes originate from two progenitor gene lineages: KIR3DX and KIR3DL. The KIR3DX lineage is represented by a single gene copy located in the center of the *LILR* gene cluster (Figure 1). The gene is fixed in most primate species, and its function is currently unknown (10). This lineage is, however, expanded in cattle, and encodes multiple inhibitory and a single activating functional KIR3DX receptor, which interact with an expanded repertoire of classical MHC molecules (11, 12). In contrast, the KIR3DL lineage expanded in primates and was diversified by duplications, deletions, and recombinations, which resulted in an elaborated *KIR* gene family. Based on their structure, ligand specificity, and/or phylogenetic analysis, the primate KIR receptors are divided into four lineages. Lineage I genes encode receptors with a D0-D2 domain configuration; lineage II (D0-D1-D2) is defined by the specificity for subtypes of HLA-A and -B in humans; lineage III includes receptors with D1-D2 and D0-D1-D2 domain configurations; and lineage V (D0-D1-D2) is represented by human KIR3DL3 and its orthologs. In the primate species studied, at least one *KIR* gene was discovered for each lineage, which indicates that gene duplication and diversification predates primate speciation. The subsequent lineage expansions are, however, species specific (Table 1).

**TABLE 1:** The number of KIR genes defined per primate species indicated per lineage.

		Lineage I			Lineage II			Lineage III			Lineage V			Total			

		Inhibitory	Activating*	Pseudogene	Inhibitory	Activating	Pseudogene	Inhibitory	Activating	Pseudogene	Inhibitory	Activating	Pseudogene	Inhibitory	Activating	Pseudogene	Total
Human	*Hosa*	2	1	0	2	1	0	3	5	2	1	0	0	8	7	2	17
Chimpanzee	*Patr*	1	1	0	1	0	0	6	3	0	1	0	0	9	4	0	13
Bonobo	*Papa*	1	1	0	3	1	0	2	0	0	1	0	0	7	2	0	9
Gorilla	*Gogo*	1	1	0	1	0	0	5	1	0	1	0	0	8	2	0	10
Bornean orangutan	*Popy*	1	1	0	1	1	0	2	3	0	1	0	0	5	5	0	10
Sumatran orangutan	*Poab*	1	1	0	1	1	0	3	3	0	1	0	0	6	5	0	11
Rhesus macaque	*Mamu*	0	1	0	31	23	0	1	0	1	1	0	0	33	24	1	58
Cynomolgus macaque	*Mafa*	0	1	0	26	30	0	1	0	1	1	0	0	28	31	1	60

*KIR2DL4 is considered an activating KIR gene.

Lineage I and V *KIR* genes have a conserved nature in all primate species examined, and comprise, respectively, *KIR2DL4* and *KIR2DL5*, and *KIR3DL3*, or a similar structure, such as *Mamu-KIR3DL20* in rhesus macaques. More extensive and species-specific expansions are reported for *KIR* genes that cluster into lineages II and III (Table 1), and the data suggest that this coincides with the evolution of their MHC class I ligands. Therefore, diversification of the lineage II and III *KIR* genes might be indirectly propelled by the adaption of the MHC class I molecules to pathogenic encounters. For hominoids, this section of co-evolution of KIR and MHC has been comprehensively reviewed by Wroblewski and colleagues (13). In short, the *MHC* gene content in great apes displays to a limited extent a variable number of *MHC-A*, *-B*, and *-C* genes per haplotype (Table 2). *MHC-C*, which originated from a duplication of an *MHC-B* gene, is fixed in all hominoids except for orangutans, where it is present on about half of the haplotypes (14). In addition, the epitopes recognized by the relevant KIR are differentially distributed across the different *MHC class I* genes (Table 2). The C1 and C2 epitopes, for example, are absent in bonobos and orangutans, respectively, whereas the A3/A11 epitope is only defined on HLA-A molecules. The hominoid MHC class I evolution is accompanied by the reduction and refinement of KIR specific for MHC-A and -B, which is reflected in their limited number of lineage II KIR receptors, whereas the emergence and fixation of MHC-C in humans, chimpanzees, and gorillas drove the expansion and specialization of lineage III KIR (Table 1) (13).

**TABLE 2:** The expansion of MHC class I genes in different primate species.

	MHC-A		MHC-B		MHC-C	
	# genes	KIR-epitopes	# genes	KIR-epitopes	# genes	KIR-epitopes
Human	1	A3/A11, Bw4	1	Bw4, C1	1	C1, C2
Chimpanzee	1	–	1	Bw4, C1	1	C1, C2
Bonobo	1	–	1	Bw4, C1	1	C2
Gorilla	1 (2*)	Bw4	1–2	Bw4, C1	1	C1, C2
Orangutan	1	–	2–4	Bw4, C1	0–1	C1
Macaque	1–3	Bw4, Bw6	1–3 (<19)	Bw4, Bw6	–	–

Indicated are the number of genes present on a single chromosome and the KIR-recognizing epitopes that may be encoded by allotypes. The frequencies of the different epitopes vary per gene and species. In macaques, on average 1–3 MHC-B genes are highly transcribed (majors), whereas the total number of genes on a single MHC haplotype can reach up to 19 copies, including low transcribed genes (minors) as well as pseudogenes (15–20). *Gorilla’s have an additional MHC-A related gene, named Gogo-Oko.

Old World monkeys, including macaques, lack an MHC-C ortholog, but instead display extensive copy number variation regarding polymorphic *MHC-A* and *-B* genes, as opposed to the fixed number of *MHC class I* genes in hominoids (Table 2) (15–18). The expression level of the different MHC-A and -B molecules, however, varies considerably in macaques. It is generally accepted that per haplotype at least a single *MHC-A* and 1 to 3 *MHC-B* genes are characterized by high transcription, and are referred to as “majors,” whereas the other *MHC class I* genes have lower transcription levels (“minors”), or may be pseudogenes. The differential transcription suggests a more classical function for the major MHC molecules, such as antigen presentation, whereas the minors might exert more specialized functions (19, 20). Only a few interactions of macaque MHC and KIR are documented, and, so far, all interactions involved lineage II KIR that recognize Bw4 and Bw6 epitopes on MHC-A and -B allotypes (Table 2) (21–25). This putative lineage II specificity for the copious macaque MHC class I repertoire coincides with an extensive ligand expansion, and, thus far, 54 and 56 different lineage II *KIR* genes have been documented for rhesus and cynomolgus macaques, respectively (Table 1) (7). Like the majors and minors for the MHC system, the *KIR* genes, may display differential expression levels, which are modulated by sequence polymorphisms and by an individual’s *MHC class I* repertoire (26–28). Lineage III *KIR* genes, which encode ligands for MHC-C in hominoids and were subject to expansion, are represented in macaques by a single gene and encodes a receptor with only the D1 extracellular domain (KIR1D). Its presence on 22% and 82% of the rhesus and cynomolgus macaque KIR haplotypes, respectively, suggests a balancing selection for this structurally modified receptor, which might execute a function other than conventional MHC recognition (29).

The maximal expression of six distinct *MHC class I* genes in most hominoids and the specialization of MHC-C as ligand for lineage III KIR is in line with their modest *KIR* gene expansion (Tables 1, 2). Macaques may harbor over 20 distinct *MHC class I* genes in one individual, of which only a few are dominantly expressed and considered to be majors. The expanded MHC repertoire in macaques probably propelled the extensive expansion and differential expression of their lineage II KIR. The balanced expansion of the *MHC* and *KIR* gene systems in primates indicates co-evolution in order to maintain a functional relation.

## Transposable Elements Facilitate Chromosomal Recombination

**FIGURE 2:**
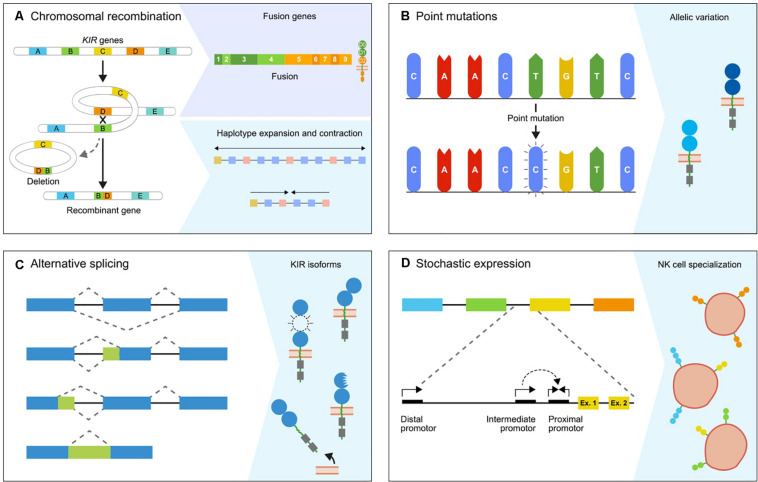
The genetic mechanisms propelling diversification. The primate KIR cluster diverged as a result of multiple molecular processes, which together modulate the *KIR* gene content and expression status. **(A)** The expansion and contraction of KIR haplotypes is mediated by chromosomal recombinations, which can introduce or remove one or multiple *KIR* genes. Occasionally, a recombination event is accompanied by the generation of a fusion gene, which functionally and structurally expands the gene repertoire. **(B)** The *KIR* genes are further diversified by point mutations in coding and non-coding regions, which generate alleles that encode receptors with different structures, localization, function, and expression. **(C)** Alternative splicing is another mechanism that has a similar impact on the function and structure of receptors. The blue and green boxes indicate exons and introns. The isoforms are generated by different splice events, which involve alternative splice sites and exon skipping. **(D)** The differential expression of subsets of KIR receptors on different NK cell clones forms another level of variation that is mediated and maintained by sequence variability in the complex promoter regions and epigenetic modifications. A conjunction of the proximal, intermediate, and distal promoters is required to induce KIR expression.

One of the mechanisms responsible for the extensive *KIR* gene diversification in macaques, and to a lesser extent in hominoids, involves chromosomal rearrangements that are accompanied by deletions and recombination, which may result in the generation of fusion genes (Figure 2A). This type of gene formation may shuffle the binding and signaling domains of different KIR receptors, thereby functionally altering the response potential of KIR family members. The dense head-to-tail arrangement of the *KIR* genes is likely to facilitate at least in part the chromosomal instability of this gene cluster. A KIR haplotype spans approximately 150 to 350 kb, depending on the number of genes present. Most *KIR* genes are separated by only 2.5 kb, as opposed to the wider haplotype configurations of more stable and less expanded gene families, such as the *LILR* gene cluster (6, 30–33). In addition, the presence of transposable elements, including Alu and LINE elements, in the intergenic and intragenic KIR sequences is another factor that further promotes genetic instability (6, 34–36). These repetitive elements are present in all primate *KIR* genes, although with species-specific variation, and drive recombination and genetic deletions (35, 37–39). For the few completely sequenced fusion *KIR* genes in humans, the chromosomal breakpoints indeed map in the intragenic transposable elements. This supports the idea that the abundant presence of transposons in the *KIR* cluster facilitates chromosome fragility, which is reflected by genetic expansion and contraction, and the formation of fusion genes (34, 40, 41). A considerable number of human fusion *KIR* genes were generated by reshuffling that involved segments of pseudogenes (34). The conservation of two pseudogenes in the human KIR repertoire, *KIR2DP1* and *KIR3DP1*, might be explained by their role in promoting recombination events. The human KIR haplotypes that include an apparent fusion gene are represented by relatively low frequencies (42–45). Positive selection of fusion entities might, however, increase their frequencies in certain populations (45). Ancient recombination events and subsequent selection might have contributed substantially to the current human KIR repertoire, but the modest expansion of the human *KIR* genes nowadays indicates limited recent recombination events. In contrast, an excessive number of recombination events are recorded in rhesus and cynomolgus macaques, with the presence of at least one fusion *KIR* gene on 42% and 49% of their haplotypes, respectively (29, 43). The abundant presence of fusion genes indicates that in these species the reshuffling of *KIR* gene segments is an ongoing process that expands the macaque KIR repertoire. Although information on the non-coding regions in the macaque *KIR* cluster is limited at present, the chromosome instability and consequential recombinations in concert with selection are likely to have driven the extensive expansion of lineage II *KIR* genes. This fast mode of evolution is further reflected in the relatively low number of orthologs that are shared between the closely related rhesus and cynomolgus macaques and their populations (29).

In all hominoids and Old World monkeys, the 5’ section of the *KIR* gene cluster is occupied by *KIR3DL3* or similar structures, which are considered framework genes and might carry out essential functions. The structure and evolutionary pathway of these lineage V *KIR* genes is a complex outcome of multiple recombination events (46). Additional chromosomal rearrangements in rhesus macaques involved the exchange of the cytoplasmic tail of *KIR3DL20* with the tails of *KIR2DL04* (lineage I) and *KIR1D* (lineage III). These recombination events are not conserved in macaque populations, which implies the relatively recent formation of novel gene entities propelled by ancient recombination hotspots (29).

Chromosomal recombination events generate genetic variability in the *KIR* gene cluster by the formation of fusion genes. Subsequent selection of these novel genes might supply an adaptive and protective strategy in the arms race with rapidly evolving pathogens.

## KIR Haplotype Diversity in Primate Species

Chromosomal rearrangements not only generate novel *KIR* gene entities by recombination but also diversify the haplotype gene content by insertions and deletions of genes (Figure 2A). In general, hominoid KIR haplotypes consist of two genomic regions that are bordered by four framework genes (Figure 1). The proximal half of the haplotype is termed the centromeric region and is defined by *KIR3DL3* to *KIR3DP1/KIRDP*, whereas the distal part, or telomeric region, ranges from *KIR2DL4* to *KIR3DL2/KIR3DL1*. Within these sections, *KIR* genes of different lineages expanded and contracted during hominoid speciation. In humans, the expansion involved lineage III *KIR* genes in their centromeric and telomeric regions, whereas expansion in chimpanzees, gorillas, and orangutans expansion took place in the same lineage in the centromeric region only (Figure 1). The human haplotype content ranges from 7 to 12 *KIR* genes, whereas the number in chimpanzee and orangutan haplotypes stretches from 5 to 11 and 5 to 10 functional *KIR* genes, respectively. In contrast to other hominoids, bonobos are characterized by a contraction of their KIR region, with only 3–7 *KIR* genes expressed on a haplotype. The shortest bonobo KIR haplotype consists of only the framework genes (47). The contracted bonobo KIR cluster coincides with a reduced nucleotide variation in their MHC class I repertoire, which might be caused by a bottleneck or pathogen-driven selective sweep after divergence from the chimpanzee’s lineage (48–51). In contrast, a highly variable *KIR* haplotype content is encountered in the macaque, with 4 to 17 functional *KIR* genes that mainly map to the telomeric region (Figure 1). The haplotype framework in macaques is less fixed than in hominoids, with only *KIR3DL20* expressed on all haplotypes, whereas *KIR2DL04* is present on 70% of the rhesus macaque haplotypes. A gene orthologous to hominoid *KIR3DL2*/*KIR3DL1* that usually marks the telomeric region is absent.

The diversified *KIR* haplotypes in hominoids and Old World monkeys stem from a primordial configuration, for which a model has been proposed by Guethlein and colleagues (35). This model describes abundant duplications and recombination events that eventually formed a conserved haplotype framework in hominoids. The previously mentioned transposable elements are likely propagating these chromosomal rearrangements that continue to mediate the diversification of haplotype configurations. One major hotspot for recombination is mapping in between the centromeric and telomeric regions, which facilitates the reorganization of the different haplotype regions. In addition, KIR haplotypes also display the insertion and deletion of one or multiple *KIR* genes propelled by unequal crossing-over, which is occasionally accompanied by the formation of a fusion gene (29, 40, 42–44). In humans, these contractions and expansions, which are mediated by double-stranded breaks at intragenic and intergenic repetitive elements, resulted in haplotypes that expressed 3 to 15 *KIR* genes (40). The short haplotypes do not express all framework genes. For instance, the deletion of *KIR2DL4* is commonly observed on genotypes defined across different populations (52, 53). Approximately 7% of the human *KIR* haplotypes are showing indications for contraction and expansion (42). Although the number of completely defined *KIR* haplotypes in other hominoids is low, several rare *KIR* configurations in chimpanzees and orangutans illustrate genetic footprints for insertion and deletion events, which is also occasionally accompanied by the formation of a fusion gene (54, 55). In macaques, only two completely sequenced haplotypes are available at present, whereas an abundant number of haplotypes are deduced at the transcription level by segregation studies (26, 29, 31, 43, 56, 57). The presence of multiple highly similar allotypes, encoded by highly similar *KIR* genes, on a single haplotype indicates an expansion by the insertion of one or more genes. Such events were recorded for 47% and 26% of the rhesus and cynomolgus macaque haplotypes, respectively (29). In contrast, a minimal *KIR* gene content and the presence of a fusion gene often are indicative of a haplotype contraction. An example of a prominent haplotype reduction in rhesus macaques involved the deletion of the complete centromeric region by an intragenic recombination of *KIR3DL20* and *KIR2DL04* (29). The variable haplotype content and the relatively high number of fusion genes indicate extensive recombination as a mechanism to diversify the macaque *KIR* gene system in a still ongoing process. This phenomenon is observed to a lesser extent for the *KIR* haplotypes in hominoids, where the process seems to have relaxed.

## The *KIR* Gene Allelic Repertoire Is Expanded by Point Mutations

Another level of variation is displayed by allelic polymorphisms, which is explained to a large extent by the occurrence of single nucleotide polymorphisms (SNP; Figure 2B). These nucleotide variations have a wide-ranging impact, and may modulate the expression level at the cell surface, ligand specificity, interaction strength, and localization of the KIR receptor. Single nucleotide variations in the extracellular D0 and D1 domains of human *KIR2DL2^∗^004* and *KIR3DL1^∗^004*, for example, retain the receptors within the cell, which might be caused by misfolding (58, 59). Polymorphisms in *KIR2DL3* alleles affect the avidity of the receptor to bind their HLA-C ligands. The low-avidity *KIR2DL3^∗^001* and the high-avidity *KIR2DL3^∗^005* only differ at three nucleotides in their D1 domain, which alters the orientation of their extracellular domains and thereby their binding strength (60). Although most KIR disease association studies determine the gene content by the presence and absence of *KIR* gene sections, and thereby lack allele-level resolution, several studies demonstrated that the functional differences of *KIR* alleles might also impact health and disease. For example, two *KIR2DL1* alleles in the African KhoeSan population evolved by single nucleotide mutations and are associated with a reduced risk for pregnancy disorders (61). Other associations demonstrated that the highly expressed *KIR3DL1* alleles are more protective against disease progression in HIV-infected individuals than lower expressed allotypes, except for the intracellularly retained *KIR3DL1^∗^004*, which is low in expression but highly protective (62–64).

A total of 1110 human *KIR* alleles are cataloged in the Immuno Polymorphism Database (IPD-KIR, release 2.9.0), whereas the number of reported alleles for different non-human primate species ranges from 521 *KIR* alleles in rhesus macaques to 5 *KIR* alleles in Bornean orangutans (IPD-NHKIR, release 1.2.0.0). These allele numbers may give a distorted view of the actual levels of polymorphism within a species due to the differential number of individuals studied. The high level of allelic polymorphism appears to be at least comparable in humans and macaques. The thoroughly documented allelic polymorphism in humans and macaques reveals a varying number of alleles per *KIR* gene, with most nucleotide variation exhibited by the framework genes (7, 29, 44). In addition, a high number of alleles were reported for certain *KIR* genes located on the telomeric haplotype region in humans (*KIR3DL1*, *KIR2DS4*) and the highly frequent inhibitory *KIR* genes in macaques (*KIR3DL01*, *KIR3DL07*). An expansion of the allele numbers for the frequently expressed *KIR* genes might indicate a continuous role in co-evolution with particular pathogens. The less common *KIR* genes, which include mostly activating KIR, vary in gene content rather than allelic polymorphism and therefore seem to execute more specialized functions and/or might be involved in the recognition of conserved ligands and peptides (7, 29, 44).

For humans, *KIR* alleles are also distinguished by SNPs in their introns (IPD-KIR, release 2.9.0) (65), which might impact, for instance, the expression level and post-transcriptional splicing. A total of 353 human *KIR* alleles can only be distinguished from the reference gene based on intronic variations (IPD-KIR, release 2.9.0), and this number is likely to be underestimated (65). Sequence data on the non-coding *KIR* gene regions are lacking for non-human primate species, but a similar extent of intronic variations might be feasible and may impact their receptor functionality. However, there are no disease or health associations reported for intronic polymorphisms within the *KIR* genes, but abundant pathological conditions are described for intronic variations in many other genes mapping elsewhere in the genome (66). For example, a SNP in the human *CYP2D6* gene is linked to a decreased expression of the functional transcript and correlates with a lower metabolic activity (67). For *HLA-DP*, a single nucleotide variation in the 3′ UTR modulates the expression level of different allotypes, which impacts the susceptibility to chronic hepatitis B virus infection (68).

Allele variation is mainly generated by synonymous and non-synonymous point mutations, and only the latter ones will impact the composition of the gene products. In sharp contrast to MHC class I polymorphisms, the allelic nucleotide variations of the *KIR* genes are evenly distributed over the coding regions. The high concentration of CpG islands located in the *KIR* gene cluster might contribute to an elevated mutation rate, as these islands are in general more prone to promote nucleotide transitions (69–71). In addition, chromosomal rearrangements are known as mutagenic events (69, 72–74). In particular, the regions that surround genomic insertions and deletions display an increased mutation rate, which might be induced by error-prone DNA replication (69, 75–77). The abundant recombination that is accompanied by insertions and deletions in the primate KIR cluster is likely to contribute to the extensive allelic KIR variation. Within two and three generations of human and macaque families studied, the birth of novel KIR alleles is described, which might further substantiate the rapid mutation rate in this gene cluster (29, 78). To our knowledge, such an event has not been recorded for the highly polymorphic *MHC class I* genes.

The variation involving *KIR* genes at the allele level impacts the interactions with their highly polymorphic MHC class I ligands, and demonstrate that point mutations contribute to a diversified *KIR* gene system. The general lack of allele level characterization in the clinic might limit the number of associations reported for *KIR* allele heterogeneity and their functional and disease-related effects. Even intronic variations might impact the KIR receptor expression and function. These few associations highlight the need to further characterize the *KIR* gene content of humans and other primate species at an allele level resolution.

## Alternative Splicing as a Mechanism for Structural Diversification

The complexity of the primate *KIR* gene cluster is further extended by alternative splicing (Figure 2C) (79–83). This post-transcriptional mechanism can generate multiple messenger RNA (mRNA) transcripts from a single gene, which are translated into different receptor isoforms. Constitutive splicing excludes the intronic sequences from the precursor mRNA (pre-mRNA) and ligates the coding exons. Alternative splicing deviates from this pattern by the use of alternative splice sites, the skipping of exons, and the retention of introns (Figure 2C) (84). The alternative splice events for human and macaque KIR transcripts are well documented, and demonstrated that both in- and out-of-frame transcripts are generated (79–83). The out-of-frame transcripts often have an early stop codon, and this results in early truncation of the transcript. Even though these out-of-frame transcripts appear as a redundant side effect of alternative splicing, it might reflect a regulatory pathway to rapidly down-regulate receptor expression. The functional impact of the in-frame generated KIR isoforms may be diverse. The skipping of exons generates transcripts that encode modified KIR isoforms, which lack one or two extracellular domains, the stem region, or the transmembrane region. These KIR isoforms probably exhibit differential binding properties or are secreted as soluble receptors (Figure 2C) (85). In-frame splice events that involve alternative splice sites might insert a partial intronic sequence into the transcript or delete a part of a coding exon. Although the functional and structural consequences of these KIR isoforms are harder to predict, they are likely to modify the receptor expression level, cellular localization, and ligand interactions.

Several splice events were frequently recorded or were defined for multiple *KIR* genes, and implicate the existence of conserved splice events that generate structurally and functionally distinct isoforms. For example, exon 4 (coding for the D1 domain) is frequently skipped from KIR3DL20 transcripts in macaques, thereby generating transcripts that encode both the complete receptor and receptors with a D0-D2 domain configuration (43, 57). This macaque isoform is termed *KIR2DL05*, as it displays an 89.5% sequence similarity with human *KIR2DL5*. Moreover, it demonstrates that alternative splicing expands the macaque KIR repertoire by generating a second two-domain structure (KIR2DL) additional to KIR2DL04. The most frequent KIR splice event in humans involved the skipping of exon 6, which encodes the stem region. Other frequent events included the skipping of exon 5 (D2 domain) and partial deletions in exons 4 and 5. These events result in isoforms that are likely to display altered binding properties, but their exact activity and localization remains elusive. Another common splice event in humans might function as a regulatory switch for expression of the 9A and 10A *KIR2DL4* alleles by restoring or disrupting the open reading frame (ORF) (79). Less frequent alternative splicing events were often found to be gene specific, and were mainly out-of-frame events that encoded for truncated receptors. Except for most exon skipping events, only a single splice event was shared between humans and macaques. This event involved a partial deletion of exon 3 (D0 domain) mediated by an alternative 5’ splice site (79). Data on the alternative splicing in other hominoids are lacking, but a similar extent of alternative splicing is likely to diversify their KIR receptors and repertoire.

The splicing of pre-mRNA not only facilitates diversification of the KIR repertoire, but might also compensate for genomic alterations that result in out-of-frame transcripts. The expression of human and macaque lineage III *KIR* genes, for example, requires the constitutive skipping of exon 3 to maintain an ORF. This exon contains a deletion of 5 bp at the genomic DNA level, which would shift the reading frame that introduces an early stop codon (79, 86). The constitutive skipping of exon 3 suggests that the expanded repertoire of human KIR2D receptors evolved from a *KIR3D* gene. The absence of a conserved 33 bp sequence in intron 2 of all human and macaque lineage III *KIR* genes might relate to the constitutive exon skipping by, for example, disrupting the spliceosome recognition site (79).

The extensive levels of alternative splicing observed in humans and macaques defines another layer of complexity for the *KIR* gene cluster. This diversifying mechanism generates structurally and functionally distinct receptor isoforms, and might be involved in the regulation of receptor expression levels. Although not all isoforms might be functional, the frequency and consistency of several alternative splicing events suggest that alternative splicing is a rapid mechanism to diversify the KIR content in hominoids and Old World monkeys.

## Differential NK Cell Populations Due to Variegated *KIR* Gene Expression

*KIR* gene plasticity is further reflected by the stochastic expression of a subset of *KIR* genes from the total gene repertoire in individual NK cells (Figure 2D). This selective transcriptional activation generates specialized NK cell populations, which express different numbers and combinations of *KIR* genes (87, 88). The stochastic KIR expression is activated during NK cell maturation, and the transcriptional pattern is maintained by the methylation of silenced *KIR* genes (28, 89). The different KIR receptor combinations are generated largely at random, but might be shaped by the individual *KIR* gene frequencies and the MHC class I repertoire. Therefore, *KIR* genes that are present on both chromosomes in heterozygous individuals, or genes that are present as two or more allotypes on a single haplotype (e.g., by duplication or gene insertion), could be expressed in a mono- and multi-allelic manner. This may generate NK cell subsets that transcribe two or more allelic copies of a certain *KIR* gene (28). Divergent expression patterns are documented for human *KIR2DL4*, which is expressed in all NK cells, and for *KIR3DL3*, which is expressed at low levels (90, 91).

The molecular regulation of *KIR* gene expression is well studied in humans, and involves multiple promoter regions in the intergenic sequences that control gene demethylation and transcription (27, 90–96). The proximal promoter is located directly in front of the first exon of a *KIR* gene and functions as a probabilistic switch (Figure 2D). Bi-directional transcription of this promoter generates forward and reverse transcripts that correlate with the activation and suppression of *KIR* gene transcription, respectively. Forward transcripts of a distal promoter are associated with activation of the proximal promoter region and appear to be required for eventual *KIR* gene expression. A third promoter upstream of the proximal promoter, also denoted as the intermediate promoter, modulates the bidirectional transcription of the proximal promoter directly or indirectly by mediating correct splicing of the forward proximal promoter transcripts (27, 94). In all human *KIR* genes, the promoter regions are highly conserved, with 91–99.6% sequence similarity. Exceptions are found for the promoters of *KIR2DL4* and *KIR3DL3*, which substantiates their diverged expression profile (94). Three types of promoter regions are defined for human *KIR2DL5*, which display considerable differences in their nucleotide sequence and transcription factor binding sites. Types I and III control variegated expression, whereas transcripts of *KIR2DL5* alleles that exhibit the type II promoter are undetectable (97, 98). These type II promoters are probably inactivated by a SNP in their Runt-related transcription factor (RUNX) transcription binding site, which is an important motif in the regulation of gene expression, and is generally conserved in all *KIR* genes (98). An identical SNP is identified in the proximal promoter of the pseudogene *KIR3DP1*, and might indicate that the inactive type II promoter is swapped to particular *KIR2DL5* alleles by chromosomal recombination (98–100). Within the *KIR* promoter regions, multiple other transcription factor binding sites are identified, which can vary per *KIR* gene and thereby contribute to differential gene expression. Allelic variations of the different transcription factor binding sites modulate the expression levels of *KIR* alleles (27, 92). For example, a *KIR2DL1* allele displayed low expression, which was associated with three SNPs in the distal promoter that generated a binding site for the Zinc finger E-box-binding homeobox 1 (ZEB1) protein (27). This transcription factor is associated with the down-regulation of IL2 expression, and might have a similar impact on the expression of this specific *KIR2DL1* allele. Just like the variation in the *KIR* gene introns, the nucleotide polymorphisms in the promoter regions are grossly undervalued, despite the direct impact on the expression of KIR alleles.

The variegated expression pattern of the *KIR* genes defines NK cell subsets, of which several are tissue resident. These NK cell populations might execute specialized functions in particular tissues that could be mediated by specific sets of KIR receptors. For example, the KIR expression profile of NK cells that were derived from the lung, liver, and uterus deviates from the expression pattern observed in peripheral blood NK cells (101–103). Expression of KIR was also established for subsets of T cells, in particular terminally differentiated CD8 + T cells, of which 30% exhibited KIR expression (104–106). The majority of these T cells dominantly express a single inhibitory or activating *KIR* gene, which is generally distinct from the *KIR* gene expression pattern on NK cells within the same individual (104). The expression pattern of NK cells and CD8 + T cells can be erased by *in vitro* treatment with a methylation inhibitor (5-azacytidine), and thereby induce the expression of formerly silenced *KIR* genes (28, 96, 107). This demonstrates that the stochastic *KIR* gene expression is maintained by methylation in both types of lymphocytes.

The variability in the promoter regions that is mainly generated by point mutations and chromosomal recombination events contributes to the diversification of NK cell subsets by the stochastic methylation of *KIR* genes. The promoter regions and epigenetic regulation of the *KIR* gene cluster in non-human primate species are less well characterized, but their stochastic expression pattern indicates a similar genetic mechanism.

## The Different Characters of Diversification in the *KIR* and *MHC* Clusters

The expansion of the primate *KIR* cluster was probably initiated by the integration of multiple retroviral elements near or in the founding *KIR* genes. Subsequent duplications were mediated by these transposable elements, and this process had an impact on the expansion of the *KIR* gene repertoire (35). These recombination events might have enhanced the mutation rate within this genomic region that generated a diverse set of *KIR* alleles, and subsequently some of these were positively selected during evolution. In the case of exons, the point mutations may affect the receptor structure, function, localization, and expression, whereas polymorphisms in the introns may enhance the level of alternative splicing by affecting existing or generating alternative splice sites. In addition, the high level of point mutations caused variation within the promoter regions, and thereby modulated the variegated expression pattern and expression level of KIR receptors. It appears that all the different molecular mechanisms are intertwined and enhanced by each other, which multiplies their diversifying impact on the primate *KIR* gene system.

The *MHC class I* gene family is considered one of the most polymorphic genomic regions in primates, but displays a different nature of diversity as compared to its KIR ligands. In hominoids, the fixed number of *MHC-A*, *-B*, and *-C* genes on a haplotype indicate low levels of recent duplications and chromosomal recombination, which is substantiated by an exceptionally low recombination rate for the *MHC class I* region (108, 109). Chromosomal rearrangements that are accompanied by the formation of an *MHC class I* fusion gene, as is determined for the *KIR* genes, is to our knowledge not known. In most hominoids, *MHC class I* polymorphism is mainly generated by point mutations in concert with a recombination of small segments. These genetic modifications are especially located in the exons encoding the peptide-binding site, and indicate a rigorous selection for a diverse array of allotypes. The functional impact is reflected in differential peptide presentation (18). Additional modification of the MHC repertoire is reflected at the transcription level by alternative splicing, which is reported for human and macaque MHC transcripts (110–114). Considering the high level of allelic polymorphism in the *HLA* genes, which may involve nucleotide substitutions that disrupt existing or generate novel alternative splice sites, one might expect abundant alternative splicing events in their transcripts. However, only a modest level of alternative splicing is demonstrated for several classical and non-classical *HLA class I* alleles, which mainly involved exon skipping that abrogated receptor surface expression (110). Specific isoforms of the non-classical *HLA-G*, however, are well known and are associated with cancer and inflammatory diseases (115–118). In contrast, alternative splicing in primate *KIR* was not limited to certain alleles, and also comprised conserved splice events that were common to multiple *KIR* genes and lineages (79). The classical MHC class I allotypes are constitutively expressed on all nucleated cells, and thereby lack a variegated expression pattern (119, 120). However, individual MHC allotypes may display a differential expression level, which is affected by sequence variation, tissue distribution, and pathogenic encounters (120, 121). In humans, the relative surface expression of HLA-A and -B is approximately ten times higher compared to HLA-C molecules (120, 122). This suggests that the *HLA-C* gene might slowly shift its main function from classical antigen presentation into the modulation of NK cell responses during infection and reproductive biology. In addition, the expression levels of different HLA-C alleles display variation, in which highly expressed allotypes correlated with a beneficial control of HIV infection (123). The differential expression pattern is also determined for the expanded MHC class I region in macaques, with only a few highly expressed MHC-A and -B allotypes (19, 124). The MHC expression levels are, however, not strictly maintained and can be modulated during infection by immune regulators such as interferon and tumor necrosis factor (TNF) (120).

The primate *KIR* and *MHC* gene families are both reflected by great complexity, and seem to co-evolve to maintain a functional relationship. The *MHC class I* diversification mainly involved allelic polymorphism in the exons encoding the peptide binding site and recombination of small segments, which is driven by the arms race with rapidly evolving pathogens. The *KIR* genes, in contrast, are diverged by haplotype expansion and contraction, random point mutations, and the generation of novel fusion genes. The expression and structural variability of the KIR receptors are further modified at the epigenetic and post-transcriptional level, whereas a similar diversification of the MHC class I molecules is limited. The conjunction of different genetic mechanisms generates an extensive plasticity for the primate *KIR* gene cluster, which seems to exceed the diversity of the polymorphic *MHC class I* genes.

## CD94:NKG2A- or KIR-Dependent Education in Different Primate Species

A comparison of the *KIR* gene system in primate species illustrates a variable degree of gene expansion, reflected in the differential expansion of gene lineages (Figure 1). This might be largely due to co-evolution with their diverse MHC class I repertoire. The variable extent of expansion, however, is emphasized by the number of functional genes per *KIR* haplotype and by the overall size of the *KIR* gene repertoire documented for a certain primate species. The extremes are represented by the heavily contracted KIR haplotypes in bonobos versus the widely expanded set of *KIR* genes in macaques (Figure 1). The flexibility to expand and contract *KIR* haplotypes and repertoires, apparently without compromising sufficient and protective immune responses, might be closely related to the nature of NK cell education in different primate species.

**FIGURE 3:**
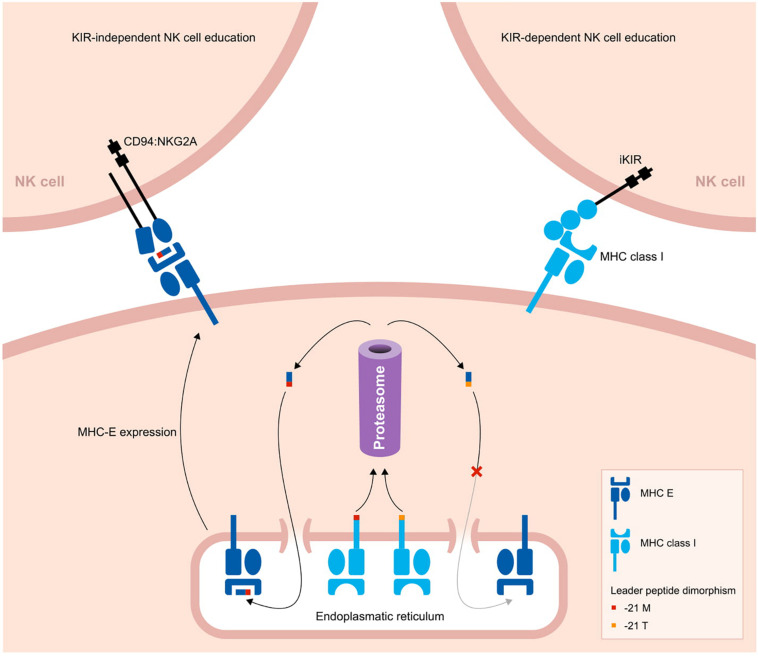
Two pathways to educate NK cells in primates. A schematic overview of two strategies to educate NK cells. The leader peptide of MHC class I molecules either contains a methionine (−21M) or threonine (−21T) residue. The -21M peptides strongly bind to MHC-E molecules, and these complexes display an increased cell surface level. Through the conserved CD94:NKG2A receptors, the MHC-E complexes educate and license NK cells. In contrast, -21T leader peptides, which are predominantly present in MHC-B allotypes of humans and chimpanzees, do not interact with MHC-E molecules. Therefore, in the presence of one or more -21T MHC class I allotypes, the cell surface level of MHC-E is decreased. In this case, NK cells are educated by MHC class I molecules that interact with their KIR ligands.

Natural killer cells require self-tolerance and a signal to activate, which are acquired through an educational process. NK cell education involves the recognition of self-MHC class I molecules or the presented peptides by at least one inhibitory NK cell receptor. Alternative educational pathways that are MHC-independent are reported, but their exact contribution to the acquiring of NK cell functions is elusive (125, 126). The MHC-dependent education is predominant and can be approached in two ways (Figure 3) (13, 127, 128). One strategy of NK cell education involves the interaction of inhibitory CD94:NKG2A NK cell receptors with the non-polymorphic MHC-E molecules, which are complexed with conserved signal peptides derived from the diversified classical MHC class I molecules (129–131). One could argue that this approach allows the immune system to scan in a crude way whether total MHC class I expression has been abrogated. In the complementary approach, however, NK cell education is established through interaction of the MHC class I molecules with polymorphic KIR receptors. This seems to reflect a more sophisticated strategy in which the immune system checks at the epitope level for a malfunctioning of MHC class I expression. KIR-dependent NK cell education is mainly conducted through the interactions of inhibitory KIR and MHC class I molecules. However, activating KIR contribute to the tuning of NK cell responsiveness by dampening NK cell activity upon MHC class I recognition (132). Currently, only for KIR2DS1 the effect on NK cell education is described. In the following sections, we mainly consider the educational impact of inhibitory KIR.

Whether the NK cells are educated by the CD94:NKG2A or KIR pathway might depend on a single nucleotide dimorphism at position 21 of the MHC class I leader sequences. Most MHC-A and -C molecules in hominoids have a methionine (−21M) residue present at this position, whereas in general this position is occupied by threonine (−21T) in MHC-B molecules. The -21M peptides strongly bind to MHC-E molecules and promote cell surface expression of MHC-E complexes (133). The presence of five or six classical MHC class I allotypes containing the -21M residue drives the NK cell education toward the more conserved MHC-E and CD94:NKG2A interactions. However, approximately 62% of human individuals display a -21T HLA-B homozygous genotype, with a variable distribution in different populations (127). In chimpanzees, -21T is near fixed in their MHC-B allotypes (13). The homozygous threonine genotype corresponds with a low MHC-E surface expression. As a consequence, human and chimpanzee NK cells are largely educated by their KIR repertoire (13, 127). In contrast, in macaque MHC-A and -B allotypes, methionine is the predominant residue at position 21 of the leader sequence, which results in an NK cell education that mostly relies on the conserved CD94:NKG2A pathway (127).

In primate species with a KIR-dependent NK cell education, one can envision that an expanded KIR repertoire may compromise NK cell activity. This might drive selection for a limited KIR expansion, as we will discuss in the next section. If this reasoning is true, the KIR-independent education of NK cells in macaques might result in an extensive expansion of their *KIR* gene system. We think that the primary function of macaque KIR is focused on the recognition and elimination of infected or malignant cells. This defense mechanism relies on the recognition of Bw4 and Bw6 epitopes, but KIR interactions are also sensitive to non-self peptides that can be presented by MHC class I molecules (134–138). A large genetic diversity of *KIR* genes provides a broader repertoire to scan all the variable MHC class I allotypes in combination with their peptides originating from pathogens. It has been proposed that up to seven distinct KIR receptors are required for successful peptide recognition (139). This optimal receptor count might even be higher when the Bw4 and Bw6 epitope specificity is considered for the different KIR allotypes. The high level of chromosomal recombination and the relatively frequent formation of fusion genes in macaques might indicate selection for a widely diversified *KIR* gene system. Considering their KIR-independent NK cell education, KIR expansion in macaques might be exempted from potential negative selection on large *KIR* gene repertoires.

However, not all macaque *KIR* haplotypes contain a large number of genes, and they even display indications for contraction by chromosomal recombination events. The formation of novel gene entities by the shuffling of head- and tail-encoding exons is achieved by recombination events, which are coherently accompanied both by contractions and expansions of *KIR* haplotypes. There might be a trade-off between the expansion of the overall KIR repertoire in a population by generating fusion genes and the contraction of KIR haplotypes in individuals. Rapid expansion and diversification generate a highly plastic macaque *KIR* gene system that appears to be maintained by selection to militate against rapidly evolving pathogens.

## KIR Haplotype Expansion and Contraction: Finding the Equilibrium

As compared to macaques, hominoids appear to have a more limited haplotype content and overall KIR repertoire (Figure 1 and Table 1). These limitations might be maintained by selective pressure on an efficient KIR-dependent NK cell education, but should be balanced with protection against infections. This balance might be reflected in the slightly variable *KIR* gene content per haplotype.

**FIGURE 4:**
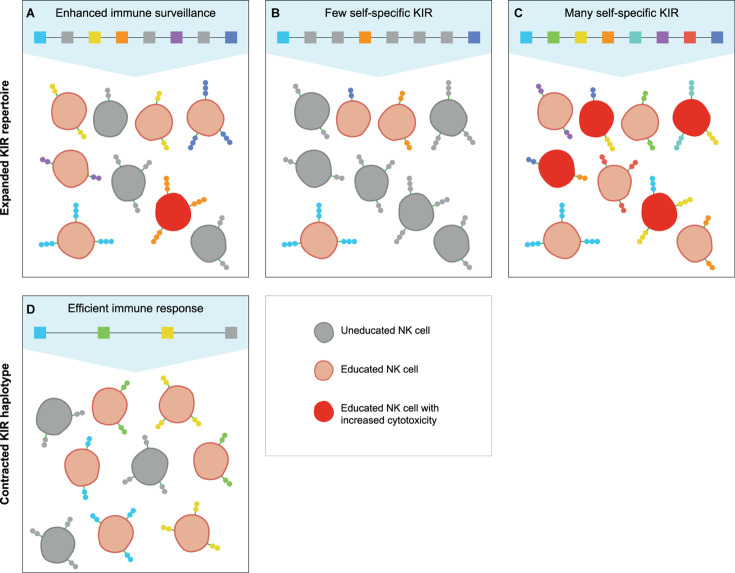
The education of NK cells by expanded and contracted KIR haplotypes. The proposed populations of NK cells that are educated (red cells) by self-specific KIR (colored boxes and receptors) on expanded and contracted haplotypes. The gray boxes represent non-self-specific KIR, and are displayed on the uneducated NK cell clones (gray cells). **(A)** An expanded KIR haplotype provides a broad MHC class I specificity, and might educate some NK cell clones through multiple self-specific receptors, which increases their cytotoxicity (dark red cells). **(B)** A large KIR repertoire with only a few self-specific KIR might lower the fraction of educated NK cells and thereby provide an insufficient immune response, **(C)** whereas abundant self-specific receptors increase the educated fraction and their cytotoxicity. **(D)** A small KIR repertoire might educate large fractions of NK cells with a limited MHC class I specificity, which might provide largely sufficient immune responses.

A large KIR repertoire is likely to provide a broad array of MHC class I specificities that may result in the education of an increased fraction of NK cells (Figure 4A). Moreover, the expression of multiple self-specific inhibitory KIR receptors by NK cell clones enhances the magnitude of their effector response (140). Although only a small population of NK cells dominantly expresses more than one inhibitory KIR receptor, an expanded KIR repertoire might enlarge this NK cell population size and elevate the strength of the NK cell response (Figures 4A,C). A potential detrimental effect of an expanded KIR haplotype might emerge if the repertoire comprises only a few or abundant self-specific receptors. On the one hand, the variegated expression of a large KIR repertoire that consists of few self-specific receptors might thin out the educated NK cell population and provide an inefficient immune surveillance (Figure 4B). Indications for a biased expression of self-specific KIR suggest modulation of the KIR expression by an individual’s MHC class I repertoire (62, 140, 141), which would ensure a more robust immune response and might compensate for a large non-self-specific KIR expansion. On the other hand, a large repertoire of self-specific KIR might enlarge the fraction of educated NK cells that display increased activity, which might be protective in infections and cancer (Figure 4C). However, elevated NK cell activity, which might be further enhanced by the expression of multiple self-specific KIR on NK cell subsets, or excessive NK cell inhibition by abundant self-specific KIR interactions are also associated with implantation failure and recurrent miscarriages (142–145). Furthermore, overactivation might desensitize NK cells and result in hyporeactivity (146), which might weaken subsequent immune responses. Therefore, a large KIR repertoire that is used in NK cell education might act as a double-edged sword that can both enhance and compromise an individual’s immune response.

In contrast, individuals that have a limited KIR haplotype rely on only one or few self-specific KIR receptors to educate their NK cells (Figure 4D). Even though a sufficient percentage of NK cells might be educated by a limited KIR repertoire, the specificity is restricted, and specialised NK cell populations might be lacking. The complete absence of NK cell education occurs in MHC class I-deficient mice, which display a near normal NK cell count with an overall reduced responsiveness (147, 148). In humans and other hominoid species, individuals that completely lack self-specific KIR are not documented. This indicates that even minimal KIR haplotypes provide education, and suggests that framework KIR receptors could play a substantial role in the NK cell education of hominoids. In addition, the chance that an individual completely lacks self-specific KIR receptors is reduced by the heterozygous nature of the *KIR* gene cluster. As far as we know, only few human and no non-human primate individuals are documented that were homozygous for their *KIR* haplotypes at an allele level (149). In a rhesus macaque family studied, one individual was assumed to be *KIR*-homozygous according to segregation. However, more detailed analysis illustrated that one *KIR* gene copy appeared to have gained point mutations that resulted in the haplotypes diverging at an allele level (29). This individual macaque possessed a largely homozygous KIR content, but did not display an impaired immune system; it also produced healthy offspring, which suggests that *KIR*-heterozygosity is not vital. However, *KIR* haplotype diversity might compensate for limited *KIR* haplotypes and improve the immune surveillance, as is also described for MHC heterozygosity (150–152).

In contrast to non-self-specific T lymphocytes, which are depleted upon a failed positive or negative selection in the thymus, uneducated NK cells are present in the peripheral blood. The relatively high level of uneducated NK cells in individuals with small or large non-self-specific KIR repertoires could affect their immune surveillance, but does not preclude an efficient immune response during infection or tumor formation. In fact, unlicensed NK cells appear to be more efficient at eradicating infected or malignant cells that persistently express MHC class I molecules or viral mimic ligands through their reactivation by cytokines or NKG2D receptors (153–155). Therefore, a fraction of uneducated NK cells in combination with a largely educated NK cell population might be more protective than a completely educated NK cell pool with broad *MHC class I* specificity.

There could be another factor, however, that limits expansion of the KIR haplotypes and gene repertoire, in addition to their role in NK cell education. In orangutans, MHC-B allotypes contain a -21M leader peptide, which would suggest education via the conserved CD94:NKG2A pathway (127). In contrast to macaques, the orangutan KIR system is not extensively expanded, and is more in line with other hominoids that display a KIR-dependent NK cell education. The emergence of MHC-C as a specialized ligand for KIR might override the dimorphism and coherent increase in MHC-E expression, and drive NK cell education via the KIR receptors. In addition, the number of characterized MHC-B molecules in orangutans is relatively low (IPD-MHC, release 3.4.0.1) (156). A larger sample group of orangutans or additional functional studies would be required to test our hypothesis for the differential KIR expansion in primate species that exert a KIR-independent or -dependent NK cell education.

Nevertheless, the diverse *KIR* haplotype content and overall gene repertoire in hominoids and Old World monkeys are likely to affect the education, activity, and function of their NK cells, but the precise effect of the haplotype expansions and contractions remains ambiguous. The equal distribution of both small and large KIR repertoires in humans and macaques indicates a balancing selection, which might be an ongoing process to achieve a haplotype equilibrium that serves differential functions, such as fighting infections and promoting successful pregnancy.

## Conclusion

The *KIR* gene system is well studied in humans, and reveals multiple mechanisms that contribute to the plasticity of this immunogenetic cluster (Figure 2). In other hominoid species, such as chimpanzees and orangutans, indications for a similar diversifying genetic toolset is evident, although robust data on some mechanisms are lacking, such as alternative splicing and variegated expression. The variability of the extensively diversified *KIR* gene cluster in macaques exceeds that observed in hominoids, with a prominent expansion of the lineage II *KIR* genes, which is largely mediated by recombination events. The rapid evolution of the *KIR* gene cluster may counteract the adaptive nature of pathogens. The species-specific diversification of the *KIR* gene cluster might be largely driven by co-evolution with their diversified MHC class I repertoire and thereby indirectly by the arms race with pathogens. In addition, a KIR-dependent or -independent NK cell education might impact the variable haplotype content and the extent of *KIR* gene expansion. Nevertheless, the different molecular mechanisms responsible for diversification of the *KIR* gene cluster are shared in Old World monkeys and hominoids, which suggests an evolutionary effort to diversify the *KIR* gene system.

## Author Contributions

JB wrote the manuscript. NG and RB edited the manuscript. All authors contributed to the article and approved the submitted version.

## Conflict of Interest

The authors declare that the research was conducted in the absence of any commercial or financial relationships that could be construed as a potential conflict of interest.
